# Recent advances in solid phase microextraction with various geometries in environmental analysis

**DOI:** 10.1039/d4ra03251a

**Published:** 2024-08-30

**Authors:** Keerthana S., Gouri Illanad, Swikriti Saket, Chiranjit Ghosh

**Affiliations:** a Department of Biotechnology, Manipal Institute of Technology, Manipal Academy of Higher Education Manipal Karnataka 576104 India chiranjit.ghosh@manipal.edu; b Department of Biotechnology, KLE Technological University Hubballi Karnataka 580021 India; c Harvard Medical School 25 Shattuck Street Boston 02115 MA USA

## Abstract

Solid phase microextraction (SPME) has emerged as a versatile sample preparation technique for the preconcentration of a broad range of compounds with various polarities, especially in environmental studies. SPME has demonstrated its eco-friendly credentials, significantly reducing the reliance on solvents. The use of biocompatible materials as a coating recipe facilitates the acceptance of SPME devices in analytical chemistry, primarily in the monitoring of environmental pollutants such as persistent organic pollutants (POPs), volatile organic compounds (VOCs), and pesticides from the various environmental matrices. During the last few years, investigators have reported an improvement in the SPME enrichment technique after changing the coating recipe, geometries, and sampling procedure from the complex matrices. Furthermore, the development of various geometries of SPME with large surface areas has enhanced the extraction efficiency of environmental pollutants. As a miniaturized sample preparation technique, SPME significantly reduces the solvent usage, suggesting a potential platform for green chemistry-based research for water, air, and soil analysis. This review article summarizes the evolution of SPME, its various modes, the application of SPME, recent innovations, and prospects for the determination of water, air, and soil pollution. The advantages and disadvantages of SPME in comparison to other extraction techniques have been discussed here. This review serves as a valuable resource for investigators working in sustainable environmental research.

## Abstract 2

Application of solid-phase microextraction technique in environmental research.

## Graphical abstract

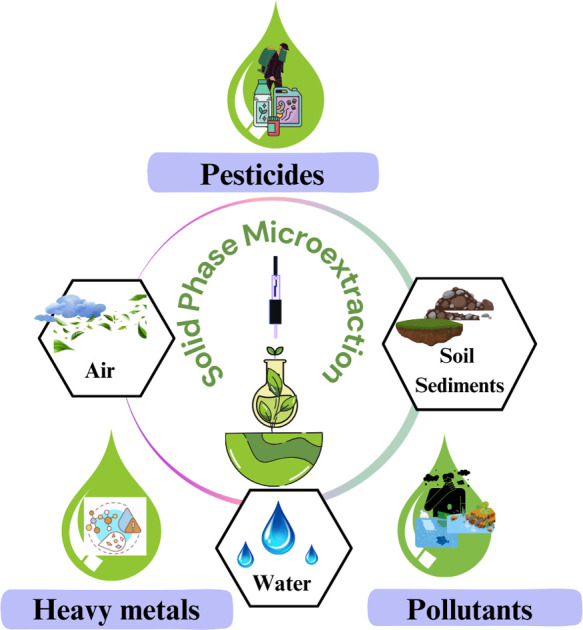

## 1. Introduction

The year 1987 marked the introduction of the concept associated with sustainable eco-development by the World Commission on Environment and Development. Therefore, it is important to preserve the natural resources for the current and future generations. A call was made for balanced development, urging that economic progress must not come at the expense of environmental protection and public health.^1^ Thus, the sustainable approach is particularly relevant for chemists, who can implement “green chemistry” principles within the laboratory and industrial settings for the optimal utilization of resources. As a result, “Green Analytical Chemistry,” or GAC, emerged in 1999 and has gained popularity, quickly becoming a commonly used term in the chemical sciences. Several research papers have been published in the same area, highlighting the importance of this theory. GAC highlights the usage of environmentally friendly sample preparation techniques at every stage of the analytical method.^2^ It also emphasizes the “3Rs” principle. This principle involves replacing the harmful solvents with greener alternatives, reducing the number and quantity of solvents used, and recycling solvents wherever possible. By following these principles, GAC minimizes the harmful impact on the environment due to the use of less solvents during the sample preparation and facilitates the widespread research using natural resources.^3^

Generally, an analytical process involves five steps: selection of sample matrix, sample preparation, separation, detection, and analysis of acquired data. These analytical processes serve as a valuable tool for ensuring stability and quality for treating the analytes.^4^ Sample preparation is considered as a crucial process since it consumes 80% of the time duration in the entire sampling process and finally influences the research output during the analysis.

Initially, the solid phase extraction (SPE) technique was derived as a powerful technique for the extraction of trace quantities of analytes present in the sample matrix. Also, SPE minimizes the use of solvents compared to the traditional liquid–liquid extraction (LLE), which still requires organic solvents and poses a concerning safety issue.^5^ Moreover, optimization of SPE for targeting the specific analytes and matrices can be challenging and time-consuming.^6^ Therefore, an alternative sample preparation technique other than SPE is required to minimize the risks associated with the traditional solvents, and the fast analysis of the samples.^7^ Accordingly, the landscape of analytical chemistry has recently been enriched by the emergence and successful application of miniaturized extraction procedures by techniques such as liquid-phase microextraction (LPME) and solid-phase microextraction (SPME). These methods can leverage the exceptional capabilities of microscopic liquid or solid phases to effectively capture and concentrate analytes of interest.^8,9^

An ideal sample preparation method should be easy to operate, economically feasible, less time-consuming, environmentally friendly, and reproducible without losing its analytes. SPME is a technique used for sample preparation and extraction of analytes from the various matrices using the principle of absorption/adsorption through the solid/liquid phases. The extraction of analytes takes place according to the equilibrium partition theory, and the subsequent desorption of the concentrated analytes in chromatographic or other analytical devices allows for the detection of those compounds. Generally, SPME has two types of conformation: static in-vessel, where the extraction phase coating is present on the outer surface, and dynamic in-flow, where the sample is made to flow inside a capillary containing the stationary extraction phase.^10^

The SPME technique requires a small number of samples during the sample preparation process, suggesting less impact on the original sample matrix than the conventional SPE technique. Researchers focused on the strategies such as safe and non-toxic extraction to tackle the challenges associated with the sample preparation. Thus, this microextraction technique combines sampling, extraction, and enrichment into a single, streamlined process. These approaches effectively extract the target compounds from complex sample matrices for efficient and eco-friendly analysis at trace levels.^11^

Due to its eco-friendly nature, SPME unsurprisingly became a popular choice for environmental analysis. Early applications focused on detecting volatile organic compounds (VOCs), persistent organic pollutants (POPs), and pesticides in water, primarily using polydimethylsiloxane (PDMS)-coated fibers. Although PDMS acts as a good adsorption material, it has a lack of selectivity for specific analytes. SPME coated with sorbent materials, including carbon nanotubes, metal–organic frameworks and molecularly imprinted polymers, offer high selectivity and act as a promising platform for water, soil and sediments, and air sample analysis.^12^ These advancements solidified SPME as a transformative tool for environmental research, promising to provide a deeper insights and more efficient method.^13^

This review article presents an overview of recent advances in SPME and its applications in detecting environmental pollutants. The article outlines the latest developments in the SPME technique that make it suitable for environmental analysis of air, water, soil, and sediments. It also discusses the strengths and limitations of the technique. Additionally, this article provides a holistic view of SPME, considering its geometries, configuration, analytical techniques and the analytes detected in various aspects of environmental applications. Researchers will find this comprehensive review highly informative as it delves deeper into the crucial role of SPME in modern analytical chemistry.

## 2. Solid phase microextraction techniques

**Fig. 1:**
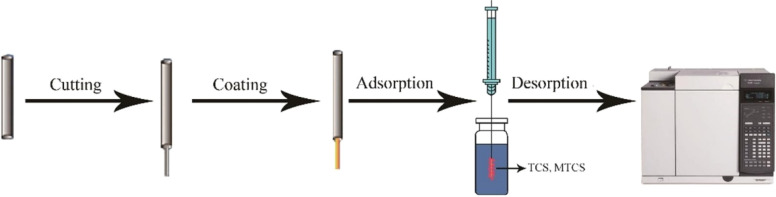
Design of SPME coated fiber by magnetic covalent organic framework nanohybrid (NiFe_2_O_4_COF) and extraction of triclosan (TCS) and methyltriclosn (MTCS).

Microextraction techniques represent an advanced approach in sample preparation of analytical chemistry.^14^ The fiber coating in SPME device acts as a selective sorbent for capturing analytes from the sample matrix.^15^ During the preconcentration stage, SPME fiber is exposed into sample matrix to enrich with polar and non-polar compounds depending on the coating materials of the fiber. Later, the fiber is dissolved into the analyzer for the quantification of the analytes.^16,17^Fig. 1 demonstrates the SPME coating procedure using covalent organic frameworks.

SPME offers two extraction approaches: equilibrium and pre-equilibrium stages. Equilibrium is a relatively slow process with high precision, whereas pre-equilibrium is comparatively faster and captures more volatile analytes. The selection of pre-equilibrium and equilibrium depends on the various influencing factors, including the volatility of analytes and sample matrix complexity.^18^

From the fundamental science point of view, SPME operates through the diffusion of the compounds from the sample matrices to coated materials as per the following equation:^19^
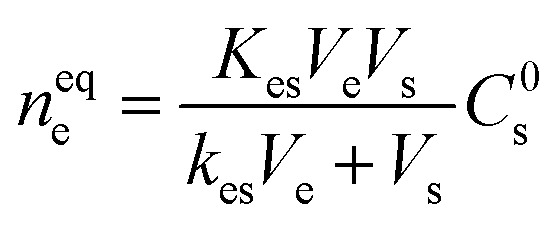
where *n*^eq^_e_ = amount of analytes extracted by the extractant.

*V*_e_ = volume of the extractant.

*V*_s_ = volume of the sample.

*C* ^0^_s_ = initial concentration of the analyte before it reaches the equilibrium.

*K*_es_ = partition coefficient or distribution coefficient.

### 2.1 SPME coating

The selection of SPME coating is important for acquiring effective extractions from different sample matrices. To select the appropriate coating materials, various properties of analytes, including polarity and volatility, analyte size and composition, should be considered. The widely used coating materials for traditional SPME applications are polydimethylsiloxane/divinylbenzene (PDMS/DVB), carboxen/polydimethylsiloxane (Car/PDMS), polydimethylsiloxane/carboxen/divinylbenzene DVB/CAR/PDMS, polyacrylate (PA), and CARBOWAX polyethene glycol (PEG).^20^ However, the applications of SPME fibres are sometimes limited due to the swelling effect of the fibers by the solvents. Therefore, the researchers further explored the alternative coating recipes to enhance the mass transfer process and enrichment factors during the analysis. Recent advances in material chemistry have facilitated the development of a wide range of sorbent coating materials for various applications. During the last few years, several coating materials, including carbon nanotubes (CNTs), graphitic carbon nitride and boron nitride nanotubes/sheets, metal–organic frameworks (MOFs), zeolitic imidazole frameworks (ZOF), covalent organic frameworks (COFs), aptamers, molecularly imprinted polymers (MOF), ionic liquids (IL), and biocompatible SPME coatings have been utilized^21^ for wide variety of applications. CNT coating is used to extract hydrophobic compounds. However, researchers utilized the functionalized CNT for extraction of polar compounds to capture pesticides, polyaromatic hydrocarbons (PAH), pharmaceuticals, *etc*.^22,23^ The successful application of SPME with CNT with polypyrrole/titanium oxide coating materials was the extraction of analytes from the water used.^24^ The coating materials are usually immobilized on the solid substrates through methods such as sol–gel chemistry, dip coating, electrolytic coating *etc.* In order to perform firm binding of coating materials on a substrate, researchers exploited suitable binders like polyacrylonitrile.^24^ To achieve effective performance using the SPME technique, it is important to optimize the coating thickness and percentage of sorbent materials within the coating recipe. Thus, the thickness of the coating may influence the time required to attain equilibrium and the desorption process during sample preparation.

### 2.2 Sampling modes of SPME

Generally, SPME offers three distinct sampling modes: direct immersion, headspace extraction, and membrane-protected extraction. Each mode caters to target the specific analyte characteristics and sample matrices during the sample extraction. Fiber SPME (Fig. 3A) presents three distinct extraction modes (DI-SPME, HS-SPME, and μ-SPME) whose efficiency is intricately linked to certain experimental parameters like agitation, salt effect, and pH.^25^

#### 2.2.1 Direct immersion solid phase micro-extraction (DI-SPME)

**Fig. 2:**
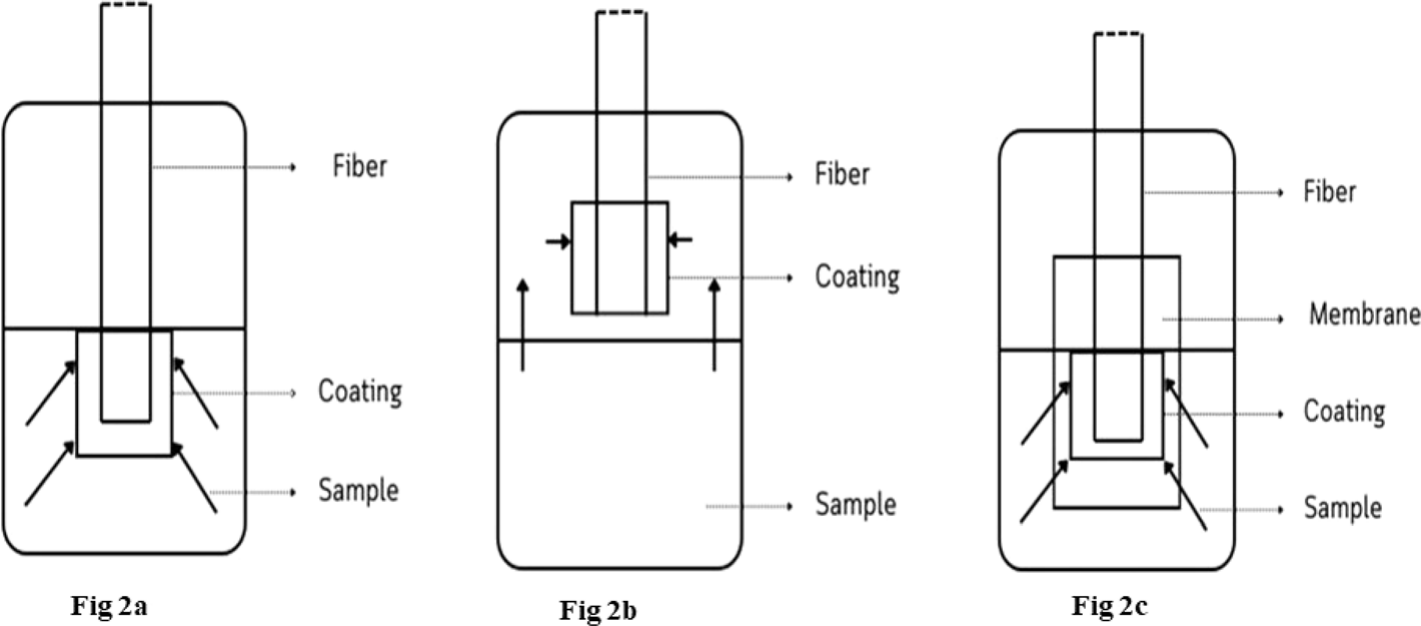
Various modes of SPME technique.

In the direct extraction mode of SPME (Fig. 2a), the coated fiber directly interacts with the sample matrix to target the analytes. This interaction enables the rapid analyte transfer from the sample to the extraction phase.^26^ However, efficient extraction depends on the diffusion limitations, particularly in the case of liquid samples, where a stagnant layer of solution can form around the fiber and it can hinder the movement of analytes. In aqueous samples, stirring or sonication disrupts the dense liquid layer surrounding the fiber, known as the “depletion zone,” and it may enhance the mass transport of analytes toward the fiber surface. Contrarily, in case of gaseous samples with inherent air flow (*e.g.*, convection), equilibration of volatile analytes might occur after certain time without the need for the agitation due to the constant movement of the air molecules.^27^ Moreover, the DI-SPME offers several advantages, including simplicity, cost savings, and adaptability to different sample matrices such as solid, liquid, and gas.^28^

#### 2.2.2 Headspace solid phase micro-extraction (HS-SPME)

Headspace extraction (Fig. 2b) is a versatile technique that can extract volatile analytes from the equilibrated vapour phase above the sample, thereby avoiding direct contact with the sample matrix.^29^ This adaptability allows the method to safeguard the fiber coating from any physical damage, even while dealing with complex matrices like non-volatile, high molecular-weight constituents like proteins.^30^

Headspace SPME has several practical applications in analyzing diverse organic compounds across matrices such as biological samples, water, soil, and air. Its unique strength lies in the minimal extraction volume, which enables the efficient concentration of trace compounds onto the fiber coating.^31^ However, certain organic compounds pose a challenge due to their strong affinity for the sample matrix. Researchers successfully overcame this hurdle after the involvement of various coating materials for strong interaction between the compounds of interest and the coating substances. The practicality of SPME is evident in its integrated design, which seamlessly transfers the absorbed compounds directly into the GC injector after the extraction and concentration. The selective absorption properties of the fiber coating act as a barrier, preventing oxygen and moisture from interfering with the GC column. This feature provides a critical advantage over direct SPME, particularly for analyzing complex matrices like greasy/oily water and human blood.^32^ A study by Rosalia *et al.* used the HS-SPME method to determine volatile compounds from mozzarella cheese following the x-ray irradiation.^33^ They observed the maximum efficiency of extraction of the volatile compounds by DVB/CAR/PDMS SPME fiber. Here, the volatolomic changes from the sample matrix may be the indicators to verify the quality of mozzarella. The study was reported to be an important technique for monitoring the quality of cheese samples.^34^ To check the analytical performance of HS-SPME fiber for the presence of untargeted metabolites in the urine matrix, researchers utilized DVB/CAR/PDMS, DVB/PDMS, CAR/PDMS, PA, and PEG fibers. They reported the DVB/CAR/PDMS as a better recipe for the extraction of compounds for the mentioned purpose. The study also reported the presence of 4-methylphenol, 4-heptanone, phenol, dimethyl disulphide, and dimethyl trisulphide compound in the urine matrix.^35^

#### 2.2.3 Membrane protection solid phase micro-extraction (μ-SPME)

Membrane-protected solid-phase microextraction (μ-SPME) is a widely used analytical technique that has successfully incorporated a diverse range of solid sorbents for capturing the analytes from both the liquid and solid matrices. This adaptability is evident in the use of various materials, including carbon nanotubes,^36^ commercially available C_18_ bonded silica,^37^ graphite fiber, and activated carbon. Unlike the conventional SPME for complete analyte extraction, μ-SPE operates at an equilibrium, which limits the maximum extractable amount.^38^ In comparison with other techniques, membrane-protected SPME (Fig. 2c) stands out by introducing a selective membrane between the fiber and the sample, effectively blocking the interferences. This unique feature is particularly beneficial when dealing with highly contaminated samples, as the protective barrier shields the fiber from the destructive effects of high-molecular-weight compounds. Though the headspace extraction offers similar protection, membrane protection extends its reach to less volatile analytes, thereby broadening its analytical scope. However, the extraction process becomes significantly slower than the direct immersion due to the additional diffusion hurdles imposed by the membrane. Fortunately, this drawback can be mitigated by employing the thin membranes and increasing the extraction temperature, which leads to a shorter analysis time.^39^

The significant sensitivity differences between the direct and headspace sampling are primarily observed in the case of highly volatile analytes. In situations where sensitivity limitations arise due to the low analyte concentration, additional steps like dispersive liquid–liquid microextraction (DLLME) can be introduced.^40^

SPME remains the most prevalent due to its simplicity of use, versatility, and compatibility with diverse analytical platforms including the gas chromatography (GC), liquid chromatography (LC) or other hypenated techniques.^40,41^

## 3. SPME configurations

### 3.1 SPME fiber

SPME offers a plethora of commercially available coatings and fiber configurations, each designed to cater to diverse analyte types and sample matrices. Depending on the convenience, the SPME encompasses various configurations: fiber, stirrer, vessel wall, suspended particle, tube, and membrane. The emerging techniques like cold SPME fiber, which utilizes a fiber that are preconditioned at a lower temperature, whereas hot-water SPME, uses high temperatures to attain an improved level of desorption, and broaden the optimization landscape for the analytes.^42^ The popular coating materials of the SPME fiber including PDMS provides for widespread applicability for polyacrylate (PA), divinylbenzene (DVB)^43^ for enhanced thermal stability, carboxen (CAR)^44^ for strong affinity towards aromatic compounds, and carbowax (CW) for capturing polar analytes. These coatings can be applied in varying thicknesses and formats, including single coatings, mixtures, and co-polymers for improving the selectivity and extraction efficiency. The diverse coating materials empower the SPME fiber applications across a broad spectrum of analytes, encompassing non-polar organic compounds (*e.g.*, BTEX, PAHs, pesticides)^45,46^ and polar organic compounds (*e.g.*, phenols, alcohols, ketones, nitroaromatics). Among the industries, Supelco (Bellefonte, PA, USA) that has pioneered SPME metal fiber assemblies featuring a metal alloy core for enhanced fiber lifetime, durability, and extraction reproducibility of the metabolic analytes.^47^

Importantly, choosing the optimal coating for a specific application is not just a step but a crucial decision in maximizing the efficacy and versatility of SPME. However, due to the limited availability of commercially available stationary phases can restrict the broad range applicability of the SPME fiber technique. Wu *et al.* developed a polypyrrole (PPY) coating, specifically tailored for extracting the polar and ionic groups of analytes.^42^ This research emphasized that the stationary phase of the fiber must exhibit high affinities for the polar analytes and should possess exceptional stability in the diverse solvent environment.^48^

An advancement in sol–gel technology has helped to revolutionize the SPME fiber design by offering a robust platform for effortless integration of organic functionalities within the inorganic, polymer-based structures within the solution phase.^49^ The reported fibers exhibited an exceptional resilience even in the harsh environments, resisting the degradation in both strong organic solvents and acidic solutions.^50^ This process involves a chemical reaction that transforms a liquid (sol) into a solid (gel) network, allowing for the development of a customized coatings. This breakthrough paves the way for the generation of diverse coatings to analyze environmental pollutants, as shown in Table 1.

**Table 1:** Applications of SPME fibers for environmental analysis

Coating	Analytes	References
PEG	BTEX, phenols, phthalic diesters, organochlorine pesticides and naphthalene congeners	51
PDMS	Benzene, toluene, ethylbenzene and xylenes (BTEX)	52
MA-MMA	Organ arsenic compounds (lewisite, methyldichloroarsine, triphenyl arsine)	53
Crown ether	Phenols, organochlorine pesticides, BTEX, acrylamides, aromatic amines	54
Graphite	BTEX	55
Benzo-15-crown-5	Volatile organic compounds	55
PPy/NiTsPc	Pesticide residue in herbal infusions	55
PDMS-PVA	Pesticides, PCB	52
LTGC	BTEX, monohalogenated benzene	56 and 57

### 3.2 In-tube SPME

**Fig. 3:**
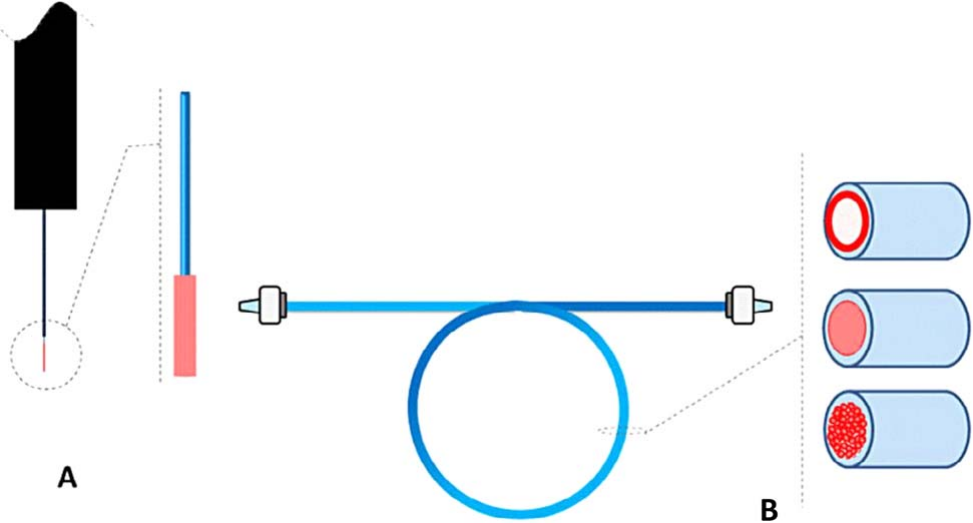
Various SPME configurations (A) fiber (B) in-tube.

In-tube SPME utilizes a segment of the capillary column as an extraction device.^58^ In-tube SPME (Fig. 3B) offers a miniaturized extraction capillary column. In accordance with the partition equilibrium, the analytes from the dilute solution are preconcentrated into the stationary stage by repetitive draw/eject phases.^59^ The extracted analytes can then be immediately transferred into the liquid/gas chromatography column.^60^ In-tube SPME has recently been used with miniaturized LC techniques to reduce the solvent usage while improving the column efficiency by providing a better compatibility. It has been utilized to assess different kinds of environmental samples. For instance, a study by Mora *et al.*^61^ developed a new method, IT-SPME-nanoLC, which is suitable for the breakdown of herbicide tribenuron-methyl (TBM) in various water sources. They observed that TBM remains stable in ambient water for weeks. However, the degradation rates vary depending on the source. In the future, the optimisation of in-tube SPME lies in developing different types of capillaries for capturing and isolating target analytes. Researchers are actively experimenting with various capillary designs like wall-coated,^62^ porous layer,^63^ sorbet packed,^64^ monolithic rod^65^ and fiber-packed capillaries.^66^

## 4. Recent applications of SPME in environmental analysis

SPME is an efficient tool for analyzing environmental pollutants across diverse matrices, including air, water, soil, and sediment, both on-site and off-site. Its popularity is evident in the numerous recent publications that have successfully applied the SPME to their environmental analysis. Tables 2–4 provides information about the wide range of applications of SPME for gaseous, aqueous, and solid matrices, respectively.

**Table 2:** Various applications of SPME for environmental gas samples analysis

Analytes	Extraction method	Capillary	Detection	References
VOCs	SPME	Micro pre-concentrator with SPME fiber	GC-MS	67
THMs (trihalo methanes)	Ambient air (SPME)	PDMS/DVB/CAR	GC-MS	43
Pesticides	ASE (accelerated solvent extraction)	Pre-concentrated SPME fiber	GC-MS/MS	46
BTEX and o-xylene	Vehicle exhaust gases (SPME)	PDMS-CAR	GC-MS	44
BTEX	Outdoor air (SPME)	Cavitand 1-coated fiber	GC-MS	45
BTEX	Laboratory air (SPME)	PDMS-coated fiber	GC-MS	68
Volatile organohalogen compounds	Workplace air (SPME)	Sol–gel-single-walled carbon nanotube/silica composite coated SPME fiber	GC-MS	69
VOCs	Air from volcanic and geothermal areas (SPME)	PDMS-DVB-CAR	GC-MS	70
VOCs	Indoor air (SPME)	PDMS-CAR	GC-MS	71
Monoterpenes	Ambient air (SPME)	PDMS-DVB	GC-FID	72
Odorous compounds	Gaseous effluents from production of poultry feathers (HS-SPME)	PDMS-DVB-CAR	GC-MS	73
Synthetic musks	Indoor air	SPE and HS-SPME	GC-MS	74
Trimethylamine	Ambient air (SPME)	PDMS-DVB	GC-FID	75
VOCs and odours compounds	Air from swine barn (SPME)	PDMS-CAR	GC-MS-O	76
Volatile organic sulphur compounds	Air from different areas of sewage treatment plan (SPME)	PDMS-CAR	GC-MS	77
Volatile carbon, sulphur and nitrogen compounds	Air from livestock operations (dynamic SPME)	PDMS-DVB-CAR	GC-MS	78

**Table 3:** Application of SPME for aqueous samples analysis

Analytes	Extraction method	Fiber	Detection	References
PCBs	SPME	Carbon nitrogen material (C_3_N_5_)	GC	79
PAH	HS-SPME	OTMS coated PG on stainless steel wire	GC-MS	80
PAH, polychlorinated biphenyls (PCBs)	SPME	PDMS-coated glass	GC-NCI-MS	81
NPAHs	SPME	ZIF-8/h-BN coated SPME fiber	GC-MS/MS	82
Organochlorine pesticides	HS-SPME	COF-CN coated fiber	GC-MS	83
Pesticides, triazines, pyrethroids	SPME	PDMS-DVB	GC-MS/SIM	84
Pesticides	DI-SPME	Polydimethylsiloxane/Divinylbenzene fiber	GC-HRMS	85
Pesticides, PAHs, BDEs, PCBs	HS-SPME	PA fiber	GC *x* gc-MS	86
Pesticide	DI-SPME	PDMS-coated fiber	GC-ECD	87
(Triclosan)TCS, (methyl triclosan) MCTS	SPME	NiFe2O4@COF-coating fiber	GC-FPD	88
OPPs (organophosphorus pesticides)	HS-SPME	G-QAS-coated fiber	TD-GC/TCD	89
Inhibition of water absorption	PI/PDMS	PDMS-coated fiber on polar PI	GC-MS	90
Methanol, ethanol, PAHs	DI-SPME	CAR/PDMS-coated fiber	GC-ECD	91
THMs	HS-SPME	PDMS-coated fiber	GC-MS/MS	92
Octocrylene (UV filters)	SPME	PDMS/DVB-coated fiber	GC-MS	93
BTEX	HS-SPME	PDMS-coated stainless steel	MS	94
PFCs (diazepam, leucine enkephalin)	SPME	RAM-coated steel and platinum wire	HPLC-MS	8
PPCPs	TFME	Mixed C18/SCX- coated on stainless steel rod	DART/Orbitrap-MS	95
Phosphoric acid esters	SBSE	PDMS-coated on still bar	GC-MS/MS	96
Organosulfur/organo arsenic compounds	HS-SPME	Methyl acrylates coated fiber	GC-NPD	97
OPFRs (TEPH, TnBP)	HS-SPME	Graphene oxide-coated fiber	GC-FID	98
*N*,*N*-Dimethylacetamide	HF-SPME	Zirconia hollow fiber and titania hollow fiber	HPLC	99
Piroxicam and diclofenac	HF-SPME	CNTs reinforced hollow fiber	HPLC	100
Phenobarbital	HF-SPME	CNTs reinforced hollow fiber	GC-MS	101
Triazines	HF-SPME	Oxidized single-walled CNTs reinforced hollow fiber	Polarography stand metrohm model 757 VA computrace	102
Lead and cadmium	HF-SPME	Ligand-assisted pseudo-stir bar hollow fiber	Atomic fluorescence spectroscopy	103
Arsenic	HF-SPME	Nanoparticles	Gas chromatography	104

**Table 4:** Applications of SPME for soil and sediment samples

Analytes	Extraction method	Capillary	Detection	References
PAHs	DI-SPME	TpPA-1 coated fiber	GC-MS	105
PAHs	CA-SPME	PDMS-coated fiber	GC-MS	106
PAHs	HS-SPME	CN-coated fiber	GC-FID	107
PAHs	HS-SPME	PDMS-DVB	GC-MS	108
PAHs	MSPME	Fe_3_O_4_ @ miSiO_2_-ph-PTSA	GC-MS	109
PAHs	DI-SPME	PMS-coated fiber	GC-MS	110
Organophosphorus	DI-SPME	PDMS-coated fiber	MS-ECD	111
BTEX	HS-SPME	CAR/PDMS-coated fiber	GC-MS	112
Organotin compounds	HS-SPME	PDMS, derivatization	GC-MS	113
Chlorophenols	HS-SPME	PA-fiber	GC-ECD	114
Herbicides	HFM-SPME	PDMS-DVB coated fiber	GC-MS	115
Fungicides	DI-SPME	PA, ultrasonic	GC-MS	116
Explosives	DI-SPME	CW-DVB	GC-ECD	117
2-Chloroethyl ethyl sulfide	HS-SPME	Acrylate/silicone co-polymer coating, sol–gel	GC	118
PCCD/F	HS-SPME	PDMS-coated fiber	GC-MS/MS	119
PCBs and PAHs	SPME	Flower-like Co_3_O_4_/C_3_N_5_ composite	GC-FID	120

### 4.1 Air samples

The monitoring of air for the presence of trace contaminants currently relies on techniques that are inhibited by inherent drawbacks compromising their usefulness and accuracy.^121^ The approaches such as grab or spot sampling in canisters or nylon bags followed by direct concentration over a sorbent bed using portable pumps and passive diffusion monitors is a potential method, but they are primarily limited due to their high operational time and expensive cost.^121^ Additionally, these methods heavily rely on sorbent materials like charcoal, silica, and polymers, whose breakthrough volumes have demonstrated a significant susceptibility in response to humidity fluctuations. Furthermore, the traditional desorption step, typically employing solvents, adds further complexity to the workflow and that can introduce contamination risks during the sample preparation. SPME presents an effective possibility for significantly enhancing the cost-effectiveness of air analysis by consolidating the initial analytical steps: sampling, extraction/concentration, and convenient delivery to the instrument. This could lead to substantial cost savings in environmental monitoring.^122^Table 2 summarizes the comprehended collection of recent SPME-based approaches for analyzing the airborne environmental contaminants.

A study by Weini *et al.* utilized an SPME technique coupled with portable GC-MS for monitoring the air pollutants.^123^ The study had several advantages compared to the use of benchtop GC-MS, including the miniaturization and determination of air pollution in remote locations where it is challenging to reach physically for sample collection. Researchers utilized MOF-801 as an active material for fabricating SPME fiber and utilized it for the estimation of the BTEX (benzene, toluene, ethylbenzene, xylene) contents from indoor air samples. Interestingly, the investigators claimed the customized SPME fiber production cost might be around $20, which is significantly more cost-effective than the commercial counterparts.^124^ A previous study by Anara Omarova *et al.* reported the feasibility of MOF-199-based SPME fiber for the monitoring of BTEX compounds from the air.^125^ The technique showed high reproducibility and repeatability with a limit of detection around 0.03 μg m^−3^. SPE-SPME-GC/MS/MS measured PAHs^126^ and antioxidants to check the indoor air quality.^127^ The combined technique of SPE and SPME was able to extract PAH contents up to 51 ng m^−3^. Apart from this, the authors reported their success for the estimation of benzothiazole (BTZ), diisobutyl-dibutyl- and di-(2-ethylhexyl)-phthalate at a low concentration range, suggesting a fast and sensitive method for extraction of multiclass compounds with various chemical moieties for indoor and outdoor air quality check.^128^ A ‘gas-cycle-assisted system’ was developed with a mini-pump, glass vial, silicone tube and speed controller and SPME fiber for quantification of organic pollutants including PAHs, polychlorinated biphenyls (PCBs), and five phthalate esters (PAEs). The proposed technique increased the extraction efficiency of semi-volatile compounds for the HS-SPME analysis.^129^ The proposed method was successful in the determination of air pollutants, including benzene, 5-methylhexanophenone, acetophenone, para-alpha-dimethylstyrene, pinane, beta-myrcene, menthol and derivatives, limonene, eucalyptol, *meta*-divinylbenzene, and *para*-ethylstyrene. Esther Borrás *et al.* reported a SPME derivatization technique for monitoring of various compounds, including aldehyde, ketones, hydroxy-aldehyde and carboxylic acid.^130^ The study demonstrated excellent efficiency with a limit of detection within the 6–100 pptv range. The main advantage of the study is that it was able to monitor the real-time trace levels of multi-oxygenated compounds in a short time. To enhance extraction efficiency, researchers utilized SPME fiber with a metal–organic frameworks (MOF) and nanoparticles-based coating for the environmental research.

### 4.2 Water samples

The application of SPME for analysis of water pollutants have been widely discussed. The fiber-retracted device, initially designed for time-weighted average air sampling, has been cleverly adapted for water. Replacing the air present in the needle with water and employing a gas-tight syringe ensures the sample integrity and minimizes the analyte carryover. These approaches demonstrate the versatility of SPME and its potential for continuous water monitoring, opening the doors for comprehensive environmental analysis.^131^

Hollow fiber SPME (HF-SPME) with commercially available and laboratory-coated capillary columns have been used to analyze triazines and arsenic in aqueous samples [37–42]. Techniques like GC-MS, GC-FID, and AFM have been used to analyze PCBs, PAHs, and arsenic, respectively, as well as other compounds in aqueous samples. In addition to the commercial SPME devices, fibers with new coating materials, including OTMS (octadecyl trichlorosilane),^80^ ZIF-8/h-BN (zeolitic imidazolate framework-8/hexagonal boron nitride),^82^ mixed C18/SCX-coated,^95^ have been used for analysis of environmental analytes in water samples. Beyond the traditional coating of fibers, researchers explored novel coating materials to enhance the selectivity and sensitivity of the SPME device. For instance, a study by Tan Lei Lu *et al.* performed a study where they utilized the pipette tip SPME technique for the monitoring of water pollutants, including sulfachlorpyridazine, sulfamethoxazole, and sulfadimethoxine from water samples.^132^ The study used the activated charcoal as an active material to fabricate the microextraction tool. It demonstrated the limit of detection around 1 ppb and good linearity of the calibration curve in the concentration range of 5–500 μg L^−1^. The advantage of the study is that it eliminates the sorbent synthesis and requires a low chemical consumption for the extraction process. Another recent study utilized chitosan cross-linked graphene oxide aerogel for coating SPME fiber and they successfully utilized it for the extraction of PAH. This study reported a low LOD value of 0.5–1000 ng L^−1^ of PAH from water matrix.^133^ Researchers utilized the direct immersion technique by polymeric ionic liquid sorbent coating of SPME fibers for the estimation of water pollutants. Karla Vargas-Berrones *et al.* proposed a low-cost technique for quantification of 4-nonylphenol in water.^134^ They utilized *N*-methyl-bis(trifluoroacetamide) (MBTFA) as a derivatizing agent on SPME fiber that can be coupled with the GC-MS for monitoring of 4-nonylphenol, an endocrine disruptor compound present in water. The study was useful for the cost-effective estimation of 4-nonylphenol. A carbon nanomaterials-based SPME device was developed for the extraction of 24 pesticides, including trifluralin, phorate, atrazine, propazine, diazinone, and napropamide.^135^ Researchers reported the presence of the 4-4-*n*-pentylphenol, 4-*n*-hexylphenol, *p-tert*-octylphenol, and nonylphenols in river waters by utilization of SPME fiber.^136^ The study exploited the stainless steel/ionic liquid SPME to monitor alkylphenols. Table 3 gives a summarized version of the analysis application of SPME in aqueous samples.

### 4.3 Soil and sediment samples

When collecting soil and sediment samples with SPME, researchers directly immerse the fiber (DI) or extract volatile compounds from the vapour emitted by the sample (HS). They often use additional techniques like sonication (sound waves), microwaves, or heating/cooling to increase extraction efficiency. Most studies rely on traditional calibration methods to quantify the extracted compounds. However, some HS-SPME analyzes of BTEX^112^ in soil utilized a specific calibration technique, which is an unique and intriguing approach to the field. Scientists developed an advanced special hollow-fiber membrane-protected SPME technique for analyzing herbicides in sewage sludge samples.^122^ Morgan W. Conrady *et al.* developed an SPME-based method for simultaneous preconcentration and analysis of geosmin and 2-methylisoborneol (2-MIB) from the soil matrix. The study also demonstrated the effectiveness of PDMS fiber for the extraction of 2-MIB.^137^

To enhance the efficiency of SPME-based extraction, researchers utilized the carbon nanospheres coated fiber for estimation of PAHs including naphthalene, acenaphthene, phenanthrene from soil and water matrices. The method reported the recoveries of PAH hydrocarbons from the water around 80–12% with a standard deviation of less than 13.9%.^138^ Further, Dina Orazbayeva *et al.* reported the feasibility of headspace SPME for monitoring epoxiconazole, metribuzin, fluroxypyr, and oxyfluorfen pesticides from soil. The study showed a promising way for determining pesticides from a complex matrix like soil.^139^ The PAHs were measured with the SPME technique with good linearity (40–4000 ng g^−1^) with low LOQ from 4.2 to 8.5 ng g^−1^. Ferrocene and five derivative compounds (ferroceneacetonitrile, ferrocenecarboxaldehyde, and benzoylferrocene, 1,1′-dimethylferrocene, and acetylferrocene) were measured by direct immersion of DVB/CAR/PDMS coated SPME fiber coupled to atomic emission detection spectrometry. The study did not find any matrix effect during the seawater sampling by SPME.^140^ However, due to the complexity of the medium, the standard addition method should be considered for the soil samples.

Further study by Xinze Wu *et al.* investigated the extraction efficiency by Zn5 cluster-coated SPME fibers for extracting ten phenolic compounds in soil samples. The work reported good linearity and low LOD using metal coordination cluster coating on SPME fibers.^141^ Qian Yan utilized the covalent organic framework-derived carbon as a coating recipe for SPME for the estimation of PAHs from soil, highlighting the applicability of this material as an SPME coating recipe for the determination of PAHs from the soil matrix^142^ which can open up a new avenue for SPME research. Table 4 explains the various applications of SPME in analyzing soil and sediment samples.

## 5. SPME for monitoring of inorganic pollutants

The detection and quantification of inorganic pollutants are critical in environmental research to ensure and monitor the quality of water, soil, and air samples. Inorganic pollutants such as heavy metals threaten human health. However, the traditional method of detecting these pollutants uses large amounts of hazardous solvents, making the process labour-intensive and cause environmental degradation. To overcome these limitations, SPME has emerged as a powerful green alternative for extracting and preconcentrating the inorganic pollutants from various matrices present in sparse quantities with high sensitivity and precision coupling with other analytical instruments.^143^

As a result of recent advancements, environmental monitoring using SPME has gained immense importance. The nano-clay demonstrated the outstanding efficiency in adsorbing and eluting Cu(ii), Cd(ii), and Pb(ii) at pH 2, using nitric acid for elution.^144^ The microextraction method exhibited high recovery percentages across a range of sample volumes from 15 mL to 70 mL. The limit of detection and limit of quantification values for Cu(ii), Cd(ii), and Pb(ii) were found to be around 1.8, 1.3, and 1.9 μg L^−1^ and 5.3, 3.9, and 5.7 μg L^−1^, respectively. The recovery percentage from various drinking water samples consistently lies within the range of 88–105%.

Magnetic solid phase microextraction (m-SPME) utilising Fe_3_O_4_ nanoparticles (NPs) was developed for the pre-concentration and analysis of cobalt and copper in natural samples such as plants, aqueous and soil samples employing microsample injection system-flame atomic absorption spectrometry (MIS-FAAS). This approach achieved recoveries of around 95% Co(ii) and 80% for Cu(ii) from 90 mL of the sample. The extraction time was around 10 minutes, with the pre-concentration factors of 180 for both Co(ii) and Cu(ii). The method yielded the low detection limits with values of 1.2 μg L^−1^ and 0.9 μg L−1 for Co(ii) and Cu(ii), respectively. The analysis was validated with the standard reference materials, including TMDW-500 drinking water, NIST 1573a tomato leaves, and NCS DC 78302 tibet soil and the method demonstrated recoveries >95% in spiking experiments.^145^

In addition, a miniaturized SPME system was designed as a simple and efficient method for concentrating cadmium (Cd) in the biological and environmental samples matrices, followed by the FAAS analysis. Then, the sample solution was treated with a complexing reagent that was drawn into the syringe and cycled through the packed tip. The analytes were extracted and sent for analysis. The technique exhibited good sensitivity and repeatability. It was successful in determining the cadmium levels available in drinking water and biological samples from the patients with the renal malfunction and healthy controls. The proposed approach was rapid and it had minimal contamination risks and was more cost-effective compared to the traditional methods. Additionally, it eases the sample preparation technique by eliminating the need for centrifugation, use of few samples and finally consumption of less solvents. This method offers a practical tool for screening and quantifying metal ions present in the various samples. The study also revealed the excess level of cadmium in the underground water and it can affect the renal function.^146^

In a further study, the researchers proposed a novel method for chromium speciation by integrating magnetism-assisted in-tube solid-phase microextraction (MA/IT-SPME) in combination with a high-performance liquid chromatography-diode array detector (HPLC-DAD).^147^ The method involved the coordination of chromium species Cr(iii) and Cr(vi) with ammonium pyrrolidine dithiocarbamate (APD) to form the complexes. The method consisted of a microextraction column containing a porous monolith doped with magnetic nanoparticles constructed within a capillary, surrounded by a magnetic coil to generate variable magnetic fields during the extraction process. The study showed that the magnetic field significantly enhanced the extraction efficiency for Cr(iii)/APD (80.4%) and Cr(vi)/APD (86.2%) complexes. The method achieved detection limits of 0.0059 μg L^−1^ for Cr(iii) and 0.0020 μg L^−1^ for Cr(vi) in water samples and 0.47 μg kg^−1^ for Cr(iii) and 0.057 μg kg^−1^ for Cr(vi) in soil samples. The technique effectively quantified Cr(iii) and Cr(vi) at trace levels in the environmental water and soil samples, demonstrating its practical applicability for the chromium speciation.

Furthermore, investigators developed a cost-effective green analytical technique for the detection of tributyltin (TBT), which is considered one of the POPs.^148^ TBT is known to impact aquatic animals in Sri Lanka. The study utilized a HS/SPME-GC/MS technique as a sensitive, simple, and solvent-free method. This technique allowed the researchers to quantify the TBT at very low levels in the various environmental samples. The study was able to quantify the TBT down to the 1 ppt level, which is below the contamination levels set by the WHO. The research work highlighted the practicality of SPME in detecting and quantifying TBT contamination in coastal environments.

The nano-clay demonstrated outstanding efficiency in adsorbing and eluting Cu(ii), Cd(ii), and Pb(ii) at pH 2, using nitric acid for elution.^144^ The microextraction method exhibited a high recovery percentage across a range of sample volumes from 15 mL to 70 mL. The limit of detection and limit of quantification values for Cu(ii), Cd(ii), and Pb(ii) were found to be around 1.8, 1.3, and 1.9 μg L^−1^ and 5.3, 3.9, and 5.7 μg L^−1^, respectively. The recovery percentage from the regular water samples consistently lies within the range of 88–105%.

In further research, a self-heating HS-SPME device was proposed using a gold-coated tungsten fiber for the detection of mercury in soil through the miniature point discharge optical emission spectrometry (μPD-OES). This method reduced the desorption time and power consumption significantly compared to the traditional external heating methods. The Au@W SPME fiber effectively preserved the mercury for long-term storage, with a sample loss of less than 5% over a one-month period. The accuracy of the device was validated using a soil-certified reference material and nine soil samples with the reported recovery rates ranging from 86% to 111%.^149^ In addition, the magnetic mesoporous carbon (Fe_3_O_4_@C, MMC) was synthesized as a coating material in magnetic dispersive solid-phase microextraction (M-dSPμE) technique to monitor the copper and lead levels in lake water, seawater, wastewater and vegetable matrices including radish, spinach, lettuce, and celery products. In the mentioned study, the various analytical parameters, including sample pH, eluent, and sample volume, were adjusted to optimize the technique.^150^

## 6. Advantages and limitations of SPME

SPME has emerged as a powerful tool in modern analytical technology, providing advantages over traditional extraction procedures. Importantly, its extraordinary automation and high-throughput capabilities reduce the general analysis time, making it an affordable option for several laboratories with large volumes of samples. Furthermore, SPME improves the accuracy and precision by reducing the possibility of error through a streamlined workflow.^151^

SPME provides various advantages over traditional methods, such as liquid–liquid extraction (LLE) in environmental analysis.^152^ To begin with, its capacity to adsorb analytes onto the sorbent fiber effortlessly allows for safe storage and transport purposes, especially for delicate or volatile compounds. This eliminates concerns about degradation during transit and facilitates field-based sampling. Moreover, SPME facilitates environmentally responsible operations by decreasing sample volume and eliminating huge solvent volumes, resulting in lower waste disposal costs. In addition, SPME enables tailored extraction by selectively interacting the analyte with the specified sorbent. This technique successfully preconcentrates the low volatile compounds and increases the range of analyzable species and enhances recovery efficiency over LLE.^153^ Finally, the selective adsorption process leads to significantly higher enrichment factors compared to LLE, resulting in improved detection limits and sensitivity, particularly for trace analysis.^154^

Despite its numerous advantages, SPME presents a few limitations that need to be carefully considered. For instance, the inter-batch and inter-manufacturer variability in fiber necessitates optimization before each analysis to achieve reliable and accurate results. Furthermore, the intrinsic fragility of the SPME fibers requires careful handling and conditioning protocols to prevent both physical damage and potential desorption of the sorbent coatings.^155^ Subsequently, gas bubble formation on the fiber surface may affect the mass transfer rates. Employing an internal standards with mass spectrometry increases the accuracy of the data but at an additional cost involved.^156^ Additionally, the limiting capacity of the fiber causes the sensitivity of SPME to peak at a specific sample volume. Selecting sorbent material may lead to matrix interferences or competitive binding among the analytes for limited binding sites that are challenging for accurate quantification, particularly in case of complex matrices. Depending on the analyte properties or complexity of the matrix, the efficiency of analyte desorption from the fiber after adsorption can vary. The sensitivity may be lower compared to some traditional techniques, especially for analytes with low affinity for the sorbent.^157^ Finally, optimizing specific analytes and matrices can be time-consuming and require expertise and it may hinder widespread adoption.^158^ By carefully addressing its limits and maximizing its benefits, SPME has enormous abilities to revolutionize analytical workflows and improve the accuracy, efficiency, and cost-effectiveness of modern research and analysis.

## 7. Conclusion

In conclusion, solid phase microextraction has emerged as a promising eco-friendly sample preparation method for environmental analysis. Its simplicity, accuracy, and integration of sampling and preconcentration in a single step have made it a popular choice among researchers. Moreover, the use of nanomaterials, metal–organic frameworks, and carbon materials as active materials has further widened the research area of this green analytical technique. As compared to traditional techniques like solid phase extraction, SPME offers several advantages for its simplicity, versatility, minimum solvent usage, and reduced sample handing process. However, several areas still require further research, such as the development of suitable methods for complex matrices, new types of coating materials, and the detection of multiple compounds by a single device. The development of an effective coating recipe for SPME will also facilitate minimising the matrix effect while working with soil and sediment samples. The development of a high-throughput SPME method is to be considered for simultaneous extraction and monitoring of pollutants from environmental matrices. Despite its advantages, researchers must also consider the possibility of incomplete extraction and loss of accuracy in complex matrices like soil samples. Therefore, further development, such as incorporating a new functional group in coating materials, is crucial to successfully integrate the SPME technique for environmental analysis. In the future, investigators may consider the current challenges and contribute to designing cost-effective and efficient SPME devices for the determination of environmental pollution levels.

## Abbreviation

AFM: Atomic force microscopy
BDE: Brominated diphenyl ethers
BDCM: Bromodichloromethane
CA-SPME: Cooling-assisted solid-phase microextraction device
COF-CN: Covalent organic framework
DBCM: Dibromochloromethane
FID: Flame ionization detector
GC/MS/MS: Gas chromatography/ion-trap tandem mass spectrometry
GC-FPD: Gas chromatography-flame photometry detector
GC-NCI-MS: Gas chromatography-negative ion chemical ionization-mass
GC-HRSM: Gas chromatography-high resolution mass spectrometry
LTGC: Low temperature glassy carbon
M-SPME: Magnetic solid phase microextraction
MSPD: Matrix solid-phase dispersion technique
MAE-HS-SPME: Microwave assisted extraction (MAE) on-line HS-SPME
MTCS: Methyltricosane
NPAHS: Nitro polycyclic aromatic hydrocarbons
PAHS: Pesticides-polycyclic aromatic hydrocarbons
PTSA: P-toluene sulfonic acid
PG: Phospho-gypsum
PA: Polyacrylate
PAN: Polyacrylonitrile
PCBS: Polychlorinated biphenyls
PAH: Polycyclic aromatic hydrocarbons
PI: Polyimide
PLE: Pressurized liquid extraction
SWCN: Single wall carbon nanotubes
TBOT: Tetra-*n*-butyl ortho titanate
TWA: Time-weighted averaging
TCS: Triclosan
THMS: Trihalomethanes
TCM: Trichloromethane
TBM: Tribromomethane
ZIF-8/h-BN: Zeolite imidazolate framework-8/hexagonal boron nitride

## Author contributions

All authors contributed to preparing the draft of the article.

## Conflicts of interest

There are no conflicts to declare.
